# Structure, Morphology and Electrical/Magnetic Properties of Ni-Mg Nano-Ferrites from a New Perspective

**DOI:** 10.3390/nano12071045

**Published:** 2022-03-22

**Authors:** Maha Mostafa, Oday Saleh, Ahmed Maher Henaish, Samir Ali Abd El-Kaream, Ryad Ghazy, Osama M. Hemeda, Ali M. Dorgham, Hanan Al-Ghamdi, Aljawhara H. Almuqrin, M. I. Sayyed, Sergei V. Trukhanov, Ekaterina L. Trukhanova, Alex V. Trukhanov, Di Zhou, Moustafa A. Darwish

**Affiliations:** 1 Physics Department, Faculty of Science, Tanta University, Al-Geish St., Tanta 31527, Egypt; maha_mostafa1990@yahoo.com (M.M.); audaysalih@gmail.com (O.S.); ahmed.henaish@science.tanta.edu.eg (A.M.H.); riad.ghazi@science.tanta.edu.eg (R.G.); omhemeda@yahoo.co.uk (O.M.H.); alimagdy1@gmail.com (A.M.D.); mostafa_ph@science.tanta.edu.eg (M.A.D.); 2 NANOTECH Center, Ural Federal University, 620002 Yekaterinburg, Russia; 3 Department of Applied Medical Chemistry, Medical Research Institute, Alexandria University, Alexandria 21526, Egypt; samir_ali852006@yahoo.com; 4 Basic Science Department, Higher Institute of Engineering and Technology, Tanta 31739, Egypt; 5 Department of Physics, College of Science, Princess Nourah Bint Abdulrahman University, Riyadh 11671, Saudi Arabia; hmalghmdi@pnu.edu.sa (H.A.-G.); ahalmoqren@pnu.edu.sa (A.H.A.); 6 Department of Physics, Faculty of Science, Isra University, Amman 11622, Jordan; dr.mabualssayed@gmail.com; 7 Department of Nuclear Medicine Research, Institute for Research and Medical Consultations (IRMC), Imam Abdulrahman Bin Faisal University (IAU), Dammam 31441, Saudi Arabia; 8 Laboratory of Magnetic Films Physics, SSPA “Scientific and Practical Materials Research Centre of NAS of Belarus”, 19, P. Brovki Str., 220072 Minsk, Belarus; sv_truhanov@mail.ru (S.V.T.); truhanov86@mail.ru (A.V.T.); 9 Laboratory of Single Crystal Growth, South Ural State University, 76, Lenin Av., 454080 Chelyabinsk, Russia; 10 Department of Electronic Materials Technology, National University of Science and Technology MISiS, 119049 Moscow, Russia; 11 Electronic Materials Research Laboratory, Key Laboratory of the Ministry of Education & International Center for Dielectric Research, School of Electronic Science and Engineering, Xi’an Jiaotong University, Xi’an 710049, China; zhoudi1220@gmail.com

**Keywords:** flash auto combustion method, nano-spinel ferrite, dielectric properties, permeability, magnetic properties, Curie temperature

## Abstract

Using the auto combustion flash method, ${\mathrm{Ni}}_{1-x}^{+2}{\mathrm{Mg}}_{x}^{+2}{\mathrm{Fe}}_{2}^{+3}O_{4}$ (x = 0, 0.2, 0.6, 0.8 and 1) nano-ferrites were synthesized. All samples were thermally treated at 973 K for 3 h. The structural analysis for the synthesized samples was performed using XRD, high-resolution transmission electron microscopy (HRTEM), and FTIR. Scanning electron microscopy (SEM) was undertaken to explore the surface morphology of all the samples. The thermal stability of these samples was investigated using thermogravimetric analysis (TGA). XRD data show the presence of a single spinel phase for all the prepared samples. The intensity of the principal peak of the spinel phase decreases as Mg content increases, showing that Mg delays crystallinity. The Mg content raised the average grain size (*D*) from 0.084 μm to 0.1365 μm. TGA shows two stages of weight loss variation. The vibrating sample magnetometer (VSM) measurement shows that magnetic parameters, such as initial permeability (*μ_i_*) and saturation magnetization (*M_s_*), decay with rising Mg content. The permeability and magnetic anisotropy at different frequencies and temperatures were studied to show the samples’ magnetic behavior and determine the Curie temperature (*T_C_*), which depends on the internal structure. The electrical resistivity behavior shows the semi-conductivity trend of the samples. Finally, the dielectric constant increases sharply at high temperatures, explained by the increased mobility of charge carriers, and decreases with increasing frequency.

## 1. Introduction

Soft ferrites have presented a broad scope in wide applications such as magneto-optical recording media and waveguides, using the disparity of several parameters such as annealing temperature and doping content [1,2,3]. Surface and quantum confinement phenomena attributable to the enormous volume to surface ratio and scale-dependent characteristics of nano-ferrites have received considerable attention. By varying such values, the desired changes in the physicochemical properties of the ferrites can be obtained [4,5].

Ferrites are suitable magnetic materials which have high degree of structural stability. So, they are utilized in some applications; in radio devices, transformer cores and switches. Nickel ferrites (NiFe_2_O_4_), also known as spinel ferrites (SF), are used in radio equipment, rod antennas, gas sensing materials and other applications [6,7,8]. Nowadays, ferrites (spinel-type) are frequently utilized in biomedical applications such as magnetic-based drug delivery systems, contrast agents in MRI (magnetic resonance imaging), and magnetic particle imaging [9].

Ni with Zn spinel ferrite nanosized samples can be prepared via spray pyrolysis [10], citrate gel [11], hydrothermal [12], sol–gel [13], microwave refluxing [14], co-precipitation [15,16], and flash auto combustion [17]. Furthermore, the mechanical ball milling method was applied to prepare nanoscale spinel ferrites. Still, this technique has certain flaws, such as rare and valuable raw materials, poor gross synthesis capabilities, and complex processes [18,19,20,21,22,23]. Ni-Zn ferrite is a relatively cheap material. Ni-Zn ferrite nanoparticles possess a high electrical resistivity; the anomalous magnetic properties are caused by the interconnected distribution of the initial Ni, Zn, and Fe cations in the crystal lattice sites are ideal for the cores of intermediate and high-frequency electromagnetic devices [24,25].

Shiv Kumar et al. [26] prepared ${\mathrm{Ni}}_{1-x}{\mathrm{Mg}}_{x}{\mathrm{Fe}}_{2}O_{4}$ (x < 0.45) by aqueous sol–gel technique and according to the magnetic measurements, they found that the prepared samples are relatively superparamagnetic materials and can be used for switching devices and as target drug delivery in the human body. Shobana et al. [27] also prepared the same composition (0.25 ≤ x ≥ 1) by the sol–gel method, and they proved that this composition could be used as an anode material for Li-ion batteries.

The novel items of this work are the impact of Mg cation doping on the structure, electrical, and magnetic properties of a series of ${\mathrm{Ni}}_{1-x}^{+2}{\mathrm{Mg}}_{x}^{+2}{\mathrm{Fe}}_{2}^{+3}O_{4}$ ferrite in order to use this ferrite in medical applications.

## 2. Materials and Methods

### 2.1. Synthesis Procedure and Method

Ferrite samples of ${\mathrm{Ni}}_{1-x}^{+2}{\mathrm{Mg}}_{x}^{+2}{\mathrm{Fe}}_{2}^{+3}O_{4}$ (x = 0, 0.2, 0.6, 0.8 and 1) were fabricated by the flash auto combustion method using nickel nitrate (Ni(NO_3_)_2_·6H_2_O), magnesium nitrate (Mg(NO_3_)_2_·6H_2_O), ferric nitrate (Fe(NO_3_)_3_·9H_2_O), and urea (CH_4_N_2_O) as a fuel. A glass rod was used to combine the metal nitrates with the urea. The mixture was heated to 80 °C with steady stirring on a hot plate until it became viscous and internal ignition occurred, resulting in brown ferrite powder formation. The final produced powder was annealed for 3 h at 700 °C.

### 2.2. Characterization Methods

A Philips model (PW-1729, Cambridge, UK) diffractometer (with *2θ* from 20° to 80°) was used to investigate the samples by X-ray diffraction. FTIR spectroscopy was used to create an FTIR spectrum in the wavenumber range from 200 cm^−1^ to 4000 cm^−1^ (Perkin Elmer 1430, Hamburg, Germany). Transmission electron microscopy (TEM) was involved in studying the microstructure of the samples (JEOL1010, Tokyo, Japan). SEM was used to investigate the morphology of the samples (JEOL JSM-6460, Tokyo, Japan). Image-J and Gatan digital micrograph software were used for calculating average pore size or pores area, the average crystallite size (*D*), and image processing. Thermogravimetric analyzer (TGA) characterizations were performed in the air with a 10 °C/min rate using a Perkin Elmer Top-Loading Series (STA-6000, Germany). A vibrating sample magnetometer (VSM) with operating system V1.6 control software was used to monitor magnetic hysteresis loops (Oxford OX8JTL, London, UK). Using the R–L–C Bridge (type BM591, Tokyo, Japan), the initial magnetic permeability (*μ_i_*), DC resistivity (*ρ*), and at various frequencies, the dielectric constant (*ε*) was calculated as a temperature function.

## 3. Results and Discussion

### 3.1. XRD (X-ray Diffraction) Discussion

**Figure 1:**
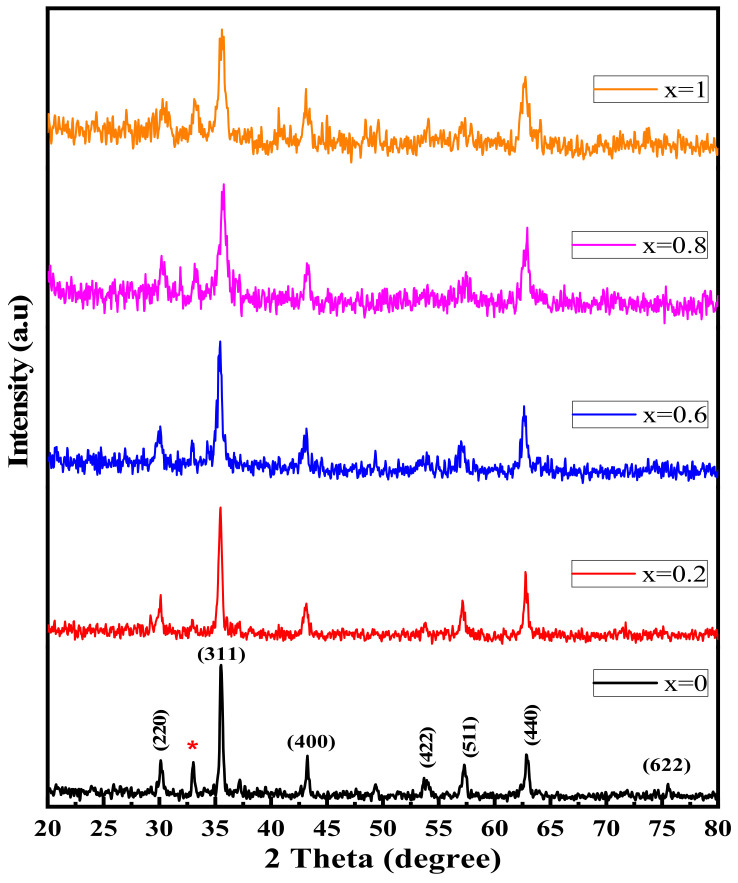
XRD patterns of ${\mathrm{Ni}}_{1-x}^{+2}{\mathrm{Mg}}_{x}^{+2}{\mathrm{Fe}}_{2}^{+3}O_{4}$ (x = 0, 0.2, 0.6, 0.8, and 1).

The XRD patterns of ${\mathrm{Ni}}_{1-x}^{+2}{\mathrm{Mg}}_{x}^{+2}{\mathrm{Fe}}_{2}^{+3}O_{4}$ (x = 0, 0.2, 0.6, 0.8, and 1) are shown in Figure 1, confirming the formation of a spinel phase according to the JCPDS standard data of Ni and Mg ferrite (PDF cards No. 742081 and 732410). The diffraction peaks are prominent and sharp, with only a small peak at around 32°, which may belong to hematite Fe_2_O_3_ [28,29,30]. The intensity of the spinel phase’s primary peak decreases as the Mg content increases. The XRD patterns confirm the polycrystalline nature, which belongs to the FCC Bravais lattice.

**Figure 2:**
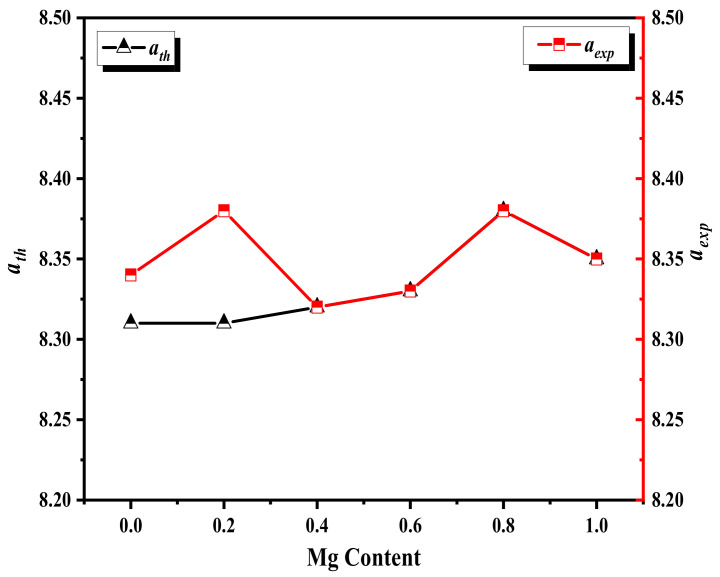
$a_{exp}$ and $a_{th}$ vs. Mg content (x = 0, 0.2, 0.6, 0.8, and 1) for ${\mathrm{Ni}}_{1-x}^{+2}{\mathrm{Mg}}_{x}^{+2}{\mathrm{Fe}}_{2}^{+3}O_{4}$.

The variation of cell parameter (*a*) with Mg content can be understood based on the ionic radii of both Mg^2+^ (0.720 Å) as well as Ni^2+^ (0.690 Å), as shown in Figure 2. Thus, the lattice expansion occurs when larger Mg ions replace the Ni ions. After x = 0.8, the Mg ions cannot be inserted in the lattice and become insoluble in Ni ferrite lattice and begin to accumulate on the lattice boundaries causing internal stress leading to the decrease of the lattice constant [31]. The shift in diffraction peak (311) occurred toward the lower diffraction angle leading to the increase of the cell parameter. The values of obtained lattice parameter are in the expected range of Ni ferrite and Mg ferrite, which are both inverse ferrites [32]. The Mg cations fill both tetrahedral (A) and octahedral (B) positions, causing Fe^3+^ cations to migrate from the (A) position to the (B) position. The four oxygen ions constituted the tetrahedral site, and six oxygen ions surrounding the B-site have to move outward along the diagonal of the lattice, causing the change in the oxygen positional parameter (*u*) as given in Table 1, which depends on the chemical composition and preparation condition and was computed using (Equation (1)) the ionic radii of the A-site ($r_{A}$), the radius of the O^−2^ ion ($r_{O}$), and lattice parameter (a) [17]:

(1) $$u=\frac{r_{A}+r_{O}}{\sqrt{3}\;a\;}+\frac{1}{4};$$

The obtained (u) values are given in Table 1, which are a little more than ideal values for the spinel ferrite (0.375 Å) but agree with the reported value of Mg ferrite [33].

The XRD data are given in Table 1 and were used to confirm the spinel phase formation by calculating the tetrahedral and octahedral radii from the supposed cation distribution from the following equations:

(2) $$r_{A}=\left(c_{AMg}r_{Mg^{+2}}+c_{AFe\;}r_{Fe^{+3}}\;\right);$$

(3) $$\;r_{B}=\frac{\left(c_{BNi}r_{Ni^{+2}}+c_{BMg\;}r_{Mg^{+2}}+c_{BFe}r_{Fe^{+3}}\right)}{2};$$

where $r_{Ni^{+2}}$, $r_{Mg^{+2}}$, and $r_{Fe^{+3}}$ are ionic radii of Ni^+2^, Mg^+2^, and Fe^+3,^ respectively, while $c_{AMg}$ and $c_{AFe\;}$ are the Mg^+2^ and Fe^+3^ ions concentration at the A-site and $c_{BNi}$, $c_{BMg}$, and $c_{BFe}$ are the Ni^+2^, Mg^+2^, and Fe^+3^ ions concentration at the B-site, respectively.

The intensities of diffraction peaks (220) and (440) provide information about the cation distribution in both A and B-sites [34]. As is well-known, the Ni ions fill the (B) position, while Mg ions fill both the (A) and (B) positions. The decrease in the intensity of diffraction peaks (220) and (440) with increasing Mg content showed a mixed cation occupancy for Mg in both the A-site and B-site [34]. Considering the preceding, the most probable distribution of cations can be concluded, which is given in Table 1.

The theoretical magnitudes of the cell parameter ($a_{th}$) can be computed taking into account the cation distribution [17] using the following:

(4) $$\;a_{th}=\frac{8}{3\sqrt{3}}\;\left[\;\left(r_{A}+r_{O}\right)+\sqrt{3}\left(r_{B}+r_{O}\right)\;\right];$$

where the radii of A-site, B-site, and oxygen are $r_{A}$, $r_{B}$, and $r_{O}$, respectively. It is noted that the value of $a_{th}$ agrees well with those experimentally (experimental lattice parameters) obtained values ($a_{exp}$), as illustrated in Figure 2.

Using Neel’s theory, the theoretical net magnetic moment ($\mu_{th}$) of the prepared samples was estimated from the expected X-ray cation distribution [2] using the following equation:

(5) $$\mu_{th}=\left|\mu_{B}\right|-\left|\mu_{A}\right|;$$

where $\mu_{A}$ and $\mu_{B}$ are the magnetic moments at A-site and B-site, respectively, and $\mu_{th}$ is calculated and given in Table 1 for all the prepared samples.

The magnetic moments used for calculating the cation distributions are Ni^+2^ = 2$\mu_{B}$, Mg^+2^ = 0$\mu_{B}$, and Fe^+3^ = 5$\mu_{B}$ [17]. The Mg content increases, the $\mu_{th}$ decreases; this is due to the decrease in the Ni^+2^ ion at the B-site, which results in a drop in the ferrite sample’s net magnetization [17].

The A-site bond length ($R_{A}$), B-site bond length ($R_{B}$), and (u) distance between ions and oxygen anion (oxygen positional parameter) at both A and B-sites were estimated [17] and given in Table 1 using the following formulas:

(6) $$R_{A}=a\;\sqrt{3}\;\left(\delta +\frac{1}{8}\right);$$

(7) $$R_{B}=a\;\sqrt{\left(3\delta^{2}-\frac{\delta }{2}+\frac{1}{16}\right)};$$

where δ is the inversion parameter at u−0.375, the bond length values steadily increase as the Mg concentration increases, which is consistent with the increase in the lattice parameter [32].

Scherer’s formula estimated the average crystallite size (*D*) from the calculated diffraction peaks width. As seen in Table 2, the *D* for all the samples decreased as the Mg concentration increased.

The theoretical X-ray density ($\rho_{x}$) is found to be larger than the bulk (measured) density ($\rho_{bulk}$), as shown in Table 2. However, the bulk density drops while the concentration of magnesium cations rises, which is explained by the lower atomic weight of Mg (24.3 at. un.) than that of Ni (58.6 at. un.), and also the decrease of density is described by the fact that the density of Mg is 1.74 g/cm^3^ which is lower than the Ni density which is equal to 8.91 g/cm^3^ [35]. The porosity (P) of the samples was computed [36] using Equation (8):

(8) $$P=1-\frac{\rho_{bulk}}{\rho_{x}}\;\left(\%\right);$$

The calculated P is given in Table 2. Due to the pores and vacancies created during the sample’s preparation, the P rises with high Mg concentration.

### 3.2. HRTEM (High-Resolution Transmission Electron Microscopy) Discussion

**Figure 3:**
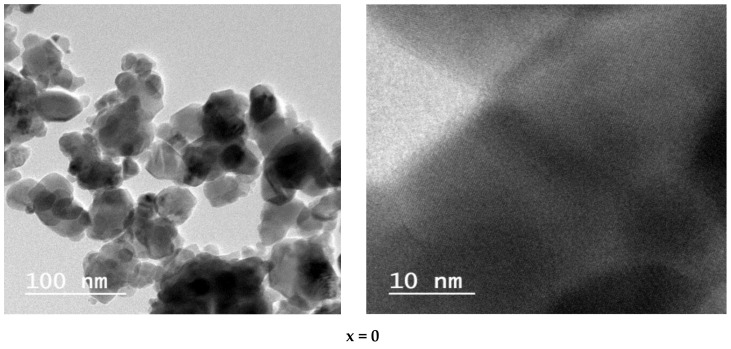

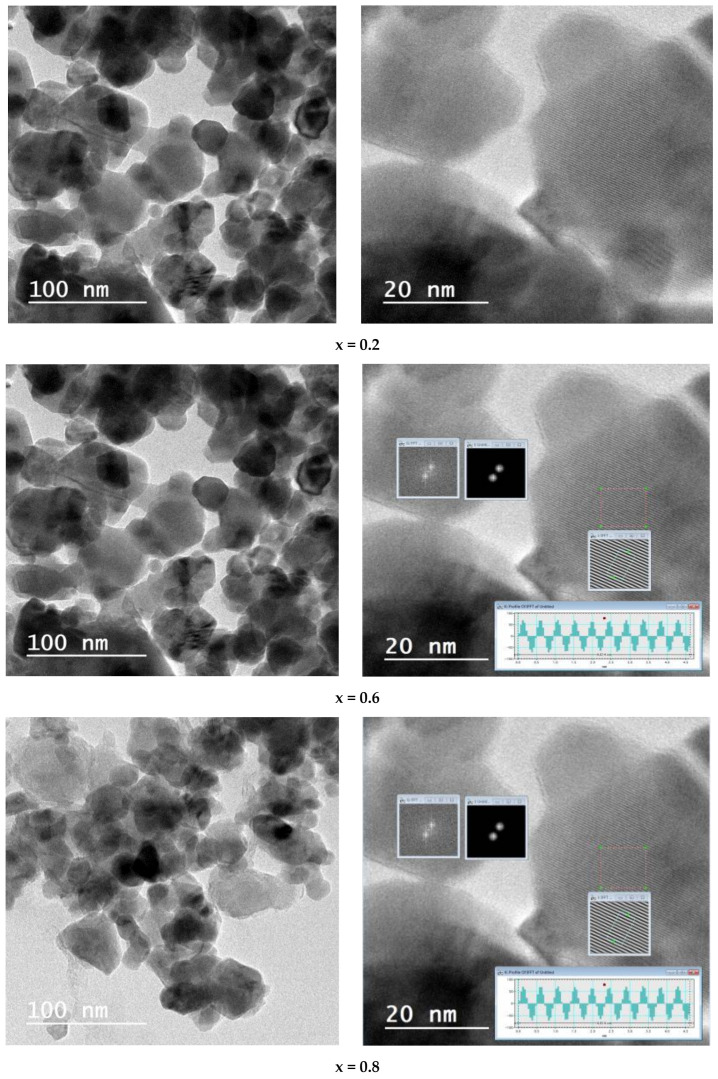

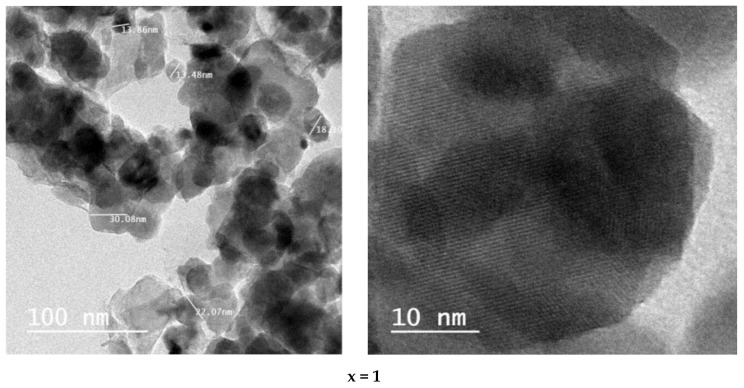
HRTEM images and the corresponding fringing spacing of ${\mathrm{Ni}}_{1-x}^{+2}{\mathrm{Mg}}_{x}^{+2}{\mathrm{Fe}}_{2}^{+3}O_{4}$ (x = 0, 0.2, 0.6, 0.8, and 1).

High-resolution TEM images of ${\mathrm{Ni}}_{1-x}^{+2}{\mathrm{Mg}}_{x}^{+2}{\mathrm{Fe}}_{2}^{+3}O_{4}$ (x = 0, 0.2, 0.6, 0.8, 1) with electron diffraction rings are illustrated in Figure 3. The TEM images display a nanocrystalline nature with some agglomeration because of the magnetic interaction between nanoparticles (NPs). The prepared samples (NPs) have a relatively circular shape (irregular), and their sizes match those calculated using Sherer’s equation from XRD measurements. The micrographs exhibit the particles’ size distribution between 17 nm and 20 nm. Corresponding lattice planes appear for each sample. The observed crystallographic value of the interplanar distance (*d*) is 2.43 Å concerning plan (311), confirming the establishment of the ferrite spinel phase. The observed crystallographic (*d*) values coincide with those gained from the XRD analysis, so the XRD and TEM confirm the spinel phase formation.

**Figure 4:**
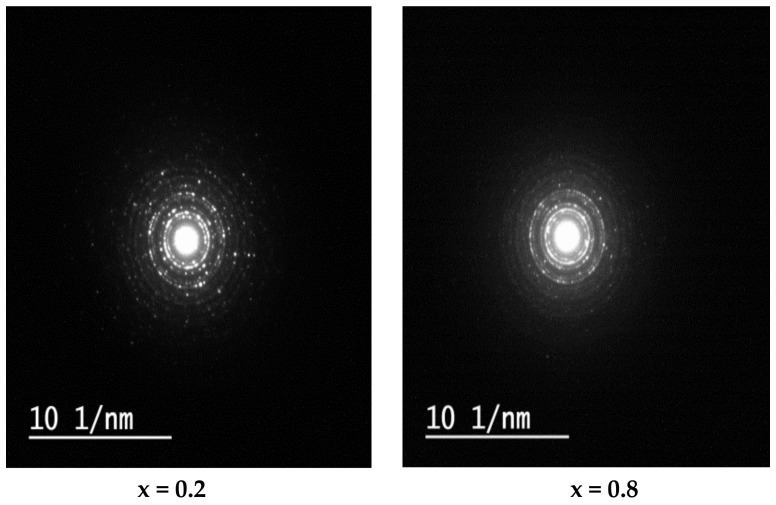
SAED patterns of ${\mathrm{Ni}}_{1-x}^{+2}{\mathrm{Mg}}_{x}^{+2}{\mathrm{Fe}}_{2}^{+3}O_{4}$ (x = 0.2, and 0.8).

Figure 4 shows the SAED (selected area electron diffraction) depiction for samples with x = 0.2 and 0.8. It was noted that the SAED depiction is composed of a set of conical halo rings that point out the nanocrystalline nature of the samples [37]. The bright spots in the halo rings represent good crystallization of the material. The crystal planes for the different circles, which correspond to different peaks of the cubic spinel phase, are defined as (220), (311), (400), (511), and (440) that appeared at the XRD pattern, which shows the consequence between electron diffraction pattern and XRD pattern [37].

### 3.3. SEM (Scanning Electron Microscopy) Discussion

**Figure 5:**
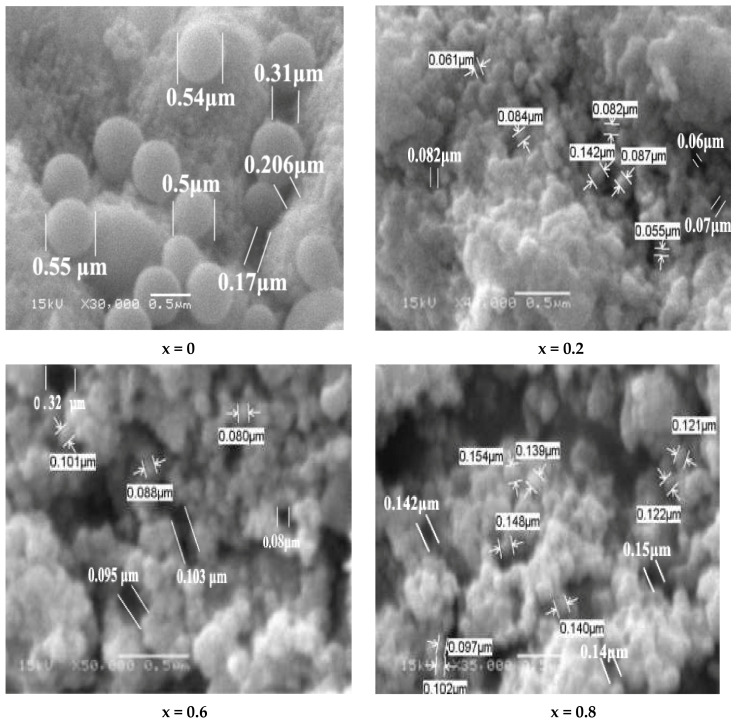

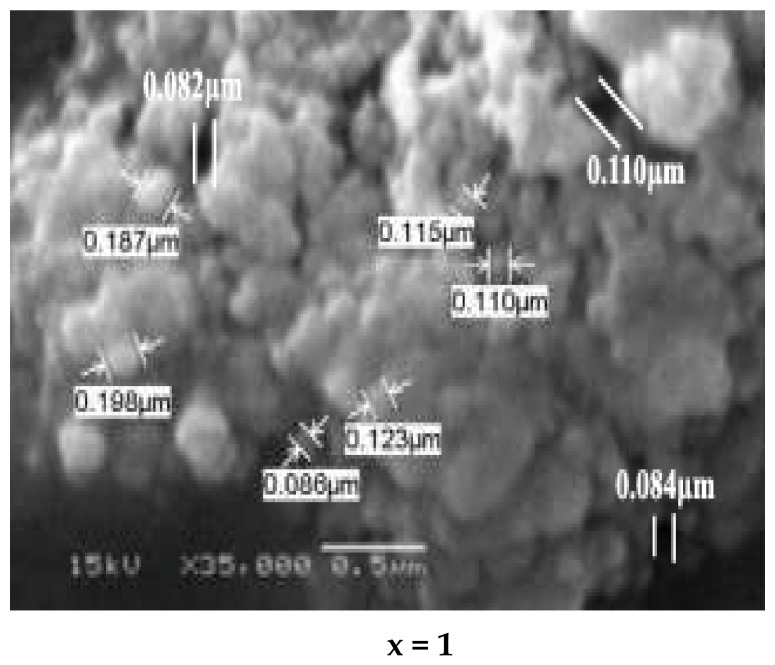
SEM micrograph of ${\mathrm{Ni}}_{1-x}^{+2}{\mathrm{Mg}}_{x}^{+2}{\mathrm{Fe}}_{2}^{+3}O_{4}$ (x = 0, 0.2, 0.6, 0.8, and 1).

**Figure 6:**
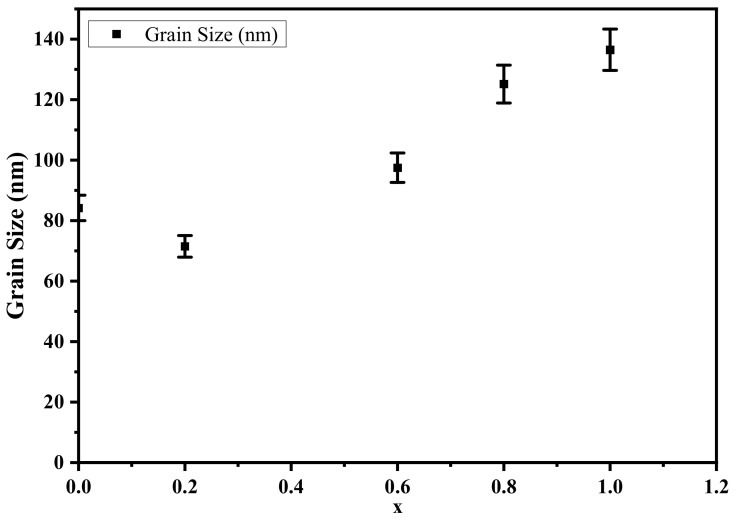
Grain size of ${\mathrm{Ni}}_{1-x}^{+2}{\mathrm{Mg}}_{x}^{+2}{\mathrm{Fe}}_{2}^{+3}O_{4}$ (x = 0, 0.2, 0.6, 0.8, and 1).

The surface morphology of the studied samples ${\mathrm{Ni}}_{1-x}^{+2}{\mathrm{Mg}}_{x}^{+2}{\mathrm{Fe}}_{2}^{+3}O_{4}$ (x = 0, 0.2, 0.6, 0.8, and 1) was inspected with scanning electron microscopy, shown in Figure 5. The grains manifest as a spherical shape with a highly compact state. An increasing trend from 84.2 nm to 13.65 nm was observed for the average grain size with increasing concentration of the Mg, as illustrated in Figure 6. A grain agglomeration occurs as the magnetic dipole moment increases by increasing Mg content because of the attractive force between grains [38]. Pores can be seen in all samples, as seen in the micrographs taken. The average grain size was determined [39] using the following equation:

(9) $$\mathrm{Grain}\;\mathrm{size}=\frac{1.5\;L}{M\;N};$$

where *N*, *L*, and *M* are the intercepts number in the SEM, the test line length, and the magnification, respectively. Furthermore, the size of the average pores was calculated and listed in Table 3; the pores’ size decreased with increasing Mg ions.

### 3.4. FTIR Spectroscopy Discussion

**Figure 7:**
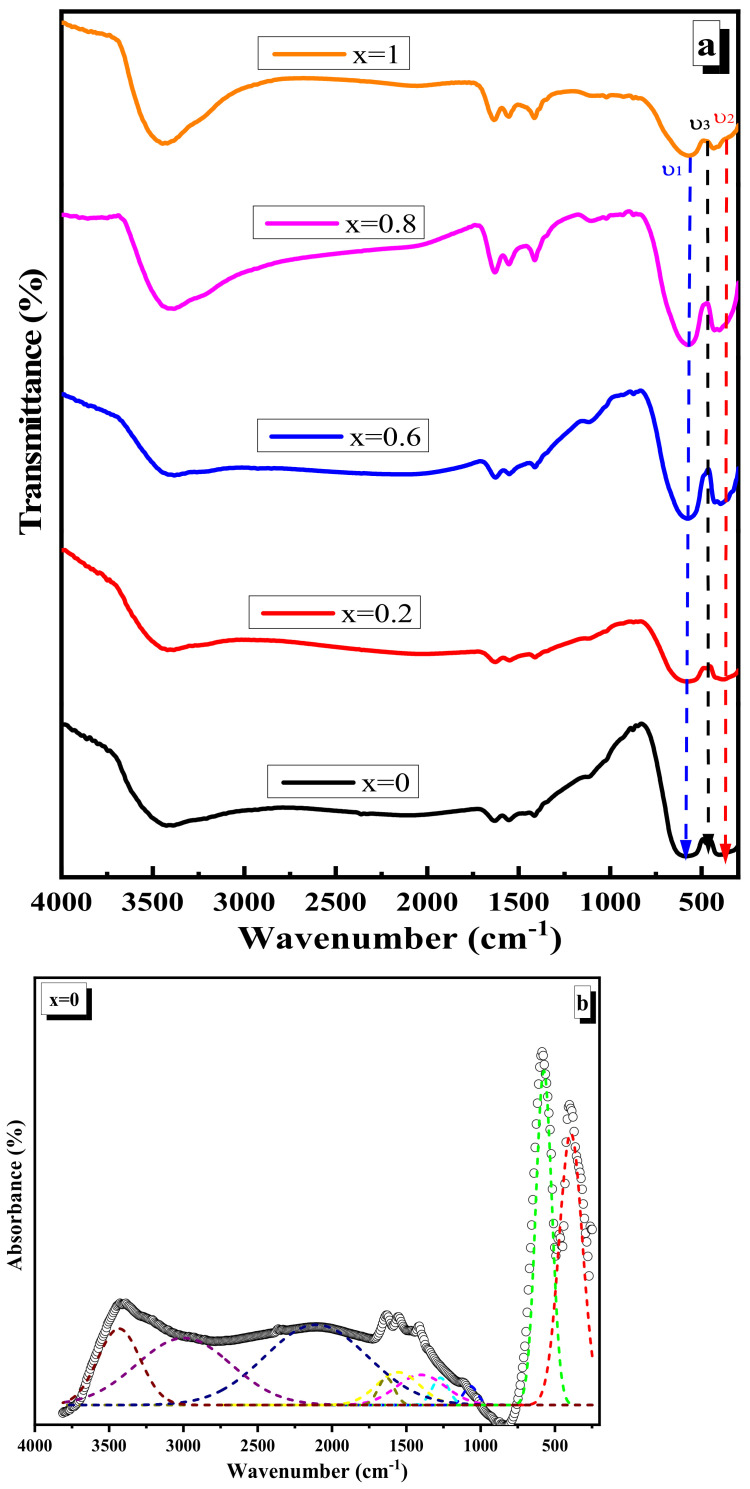

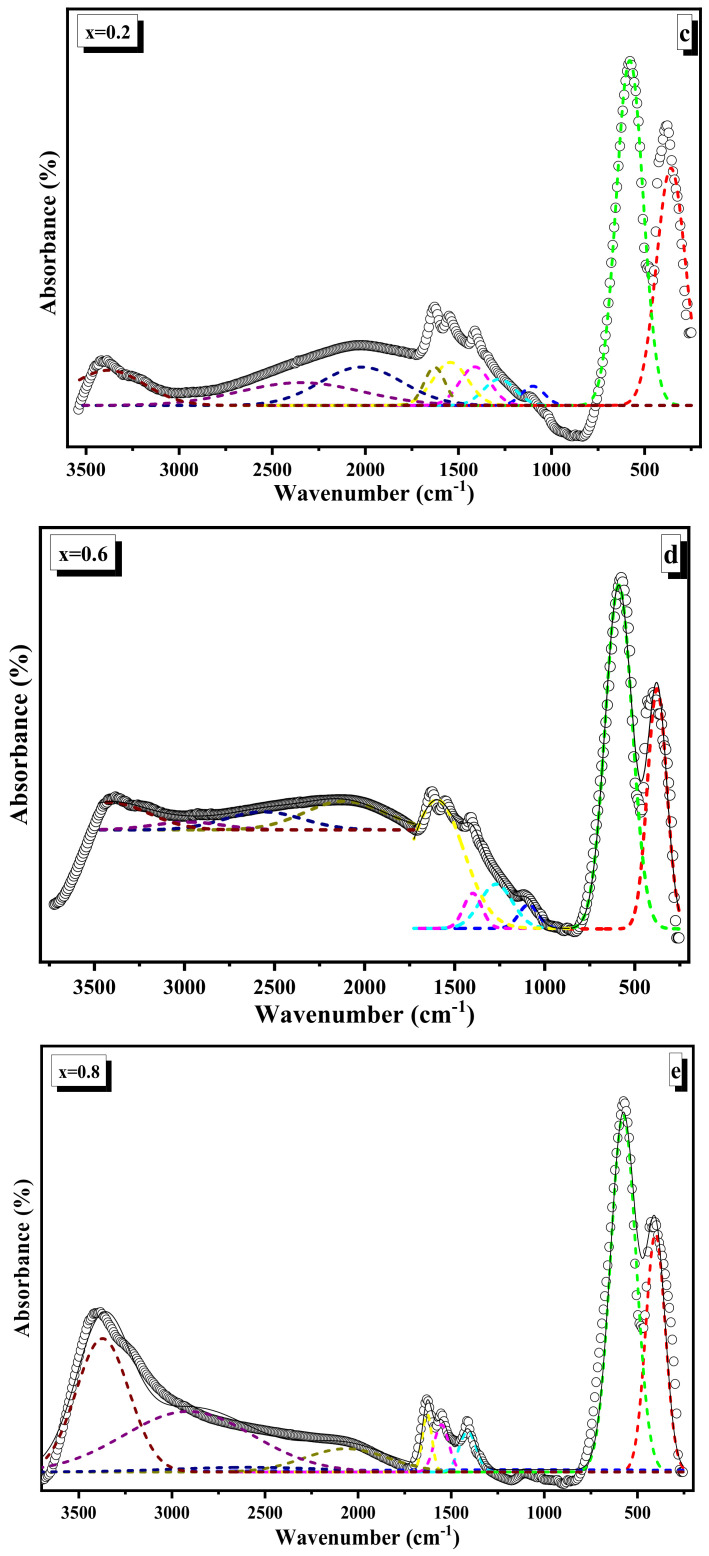

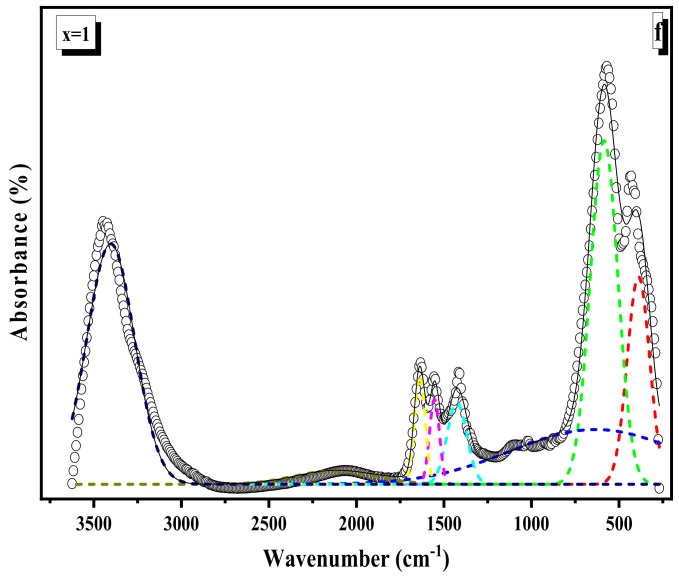
(**a**) FTIR spectra of ${\mathrm{Ni}}_{1-x}^{+2}{\mathrm{Mg}}_{x}^{+2}{\mathrm{Fe}}_{2}^{+3}O_{4}$ (x = 0, 0.2, 0.6, 0.8, and 1). (**b**) Deconvolution of FTIR absorbance spectra of ${\mathrm{Ni}}_{1-x}^{+2}{\mathrm{Mg}}_{x}^{+2}{\mathrm{Fe}}_{2}^{+3}O_{4}$ (x = 0). (**c**) Deconvolution of FTIR absorbance spectra of ${\mathrm{Ni}}_{1-x}^{+2}{\mathrm{Mg}}_{x}^{+2}{\mathrm{Fe}}_{2}^{+3}O_{4}$ (x = 0.2). (**d**) Deconvolution of FTIR absorbance spectra of ${\mathrm{Ni}}_{1-x}^{+2}{\mathrm{Mg}}_{x}^{+2}{\mathrm{Fe}}_{2}^{+3}O_{4}$ (x = 0.6). (**e**) Deconvolution of FTIR absorbance spectra of ${\mathrm{Ni}}_{1-x}^{+2}{\mathrm{Mg}}_{x}^{+2}{\mathrm{Fe}}_{2}^{+3}O_{4}$ (x = 0.8). (**f**) Deconvolution of FTIR absorbance spectra of ${\mathrm{Ni}}_{1-x}^{+2}{\mathrm{Mg}}_{x}^{+2}{\mathrm{Fe}}_{2}^{+3}O_{4}$ (x = 1).

The FTIR spectra of the nano ferrite samples ${\mathrm{Ni}}_{1-x}^{+2}{\mathrm{Mg}}_{x}^{+2}{\mathrm{Fe}}_{2}^{+3}O_{4}$ (x = 0, 0.2, 0.6, 0.8, and 1) are given in Figure 7a. The absorption spectra show two major absorption bands, around 580 cm^−1^, and 382 cm^−1^. The first absorption band specified the stretching vibration of Fe^+3^–O^−2^ at the A-site, whereas the second absorption band was particular to the B-site bond vibration [40]. The peaks around 1414 cm^−1^ represent the vibration of O–H [41]. By contrast, the bands that appear at around 1600 cm^−1^ and 3500 cm^−1^ are caused by the bending vibration mode of water molecules and the stretching vibration mode of H–O–H [17]. Variation in the Fe^+3^ population results in numerous modes of vibration at both the A and B-sites, resulting in changes in the absorption band and widening [38,42].

From the X-ray analysis, the bond length of Fe^+3^–O^−2^ at the A-site increases, leading to the decrease of stretching vibration frequencies of the tetrahedral complex, as shown in Table 4. This trend suggests that both the A and B-sites occupy the Mg ion, which results in the displacement of Fe^3+^ ions away from the oxygen ion, i.e., increases the Fe^+3^–O^−2^ distance on both A and B-sites [32]. X-ray results confirmed this discussion.

The force constant (*F*) for Fe^+3^–O^−2^ bonds at both the A and B-sites was estimated using the formula *F* = 4*π*^2^*C*^2^*μv*^2^, where *v* is the frequency, *μ* is the reduced mass, and *C* is the speed of light in cm/sec [43]. As seen in Table 4, the F of the A-site decreases as the Mg level increases. This is due to the A-site’s vibrating frequency decreasing as the Mg amount increases. The F of the B-site decreases as the Mg content increases up to x = 0.2 and then increases, mimicking the absorption frequency *ν*_2_.

Due to the overlapping absorption bands, the experimental spectra have to be deconvolved. This technique was carried out with the use of a Gaussian function. The deconvolution approach enabled more accurate identification of FTIR bands in experimental spectra. Figure 7b–f shows an example representation of the deconvoluted FTIR spectrum of the ferrite sample with ten Gaussian components. Table 5 shows the peak positions and relative area (%) assignments.

### 3.5. TGA (Thermogravimetric Analysis) Discussion

**Figure 8:**
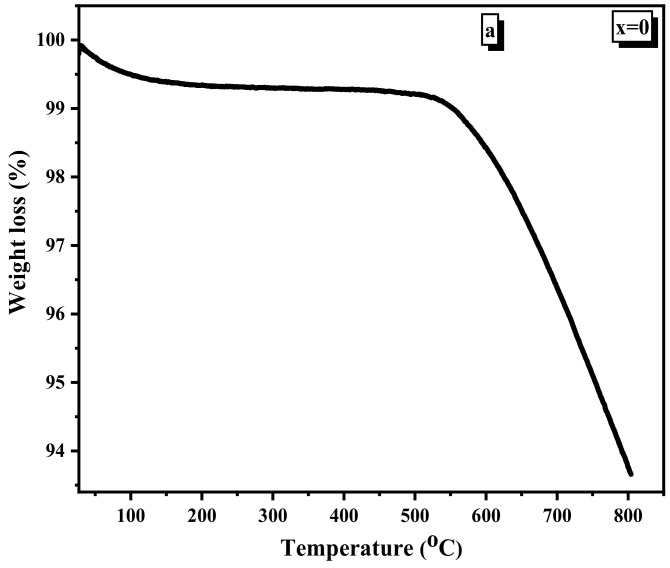

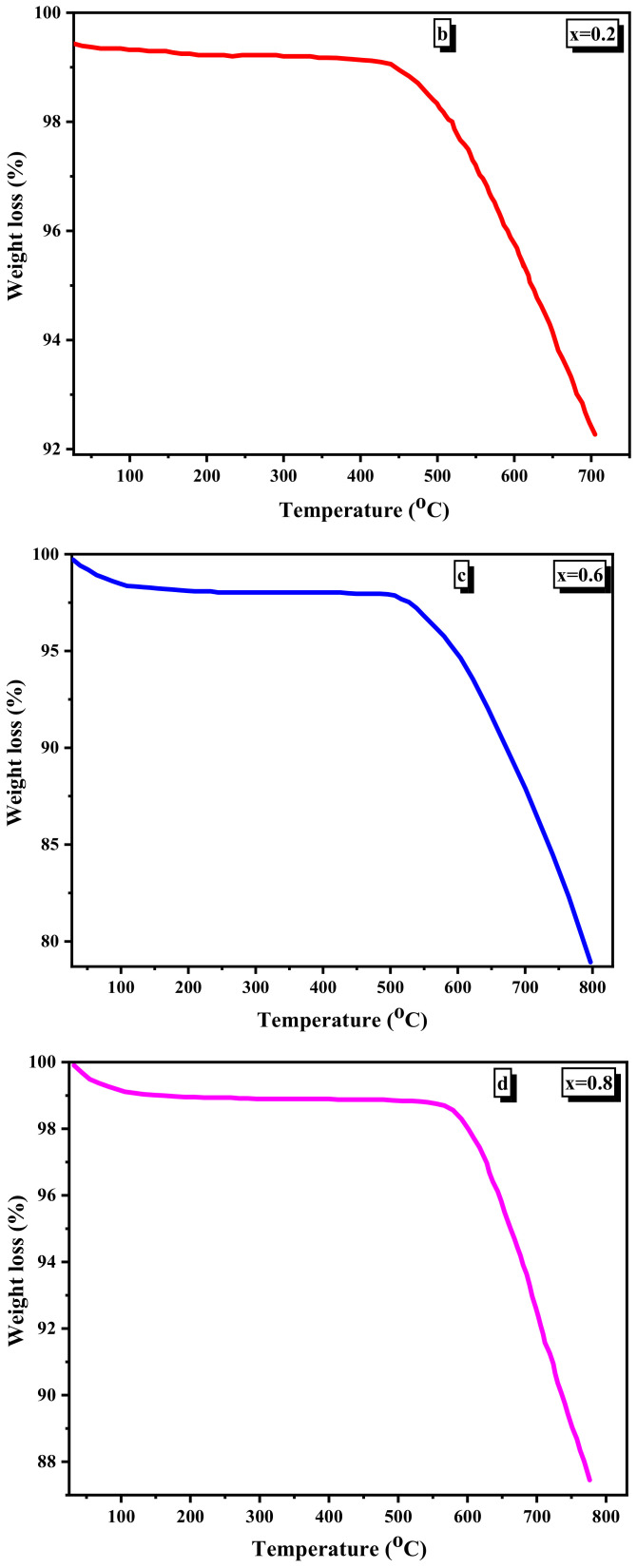

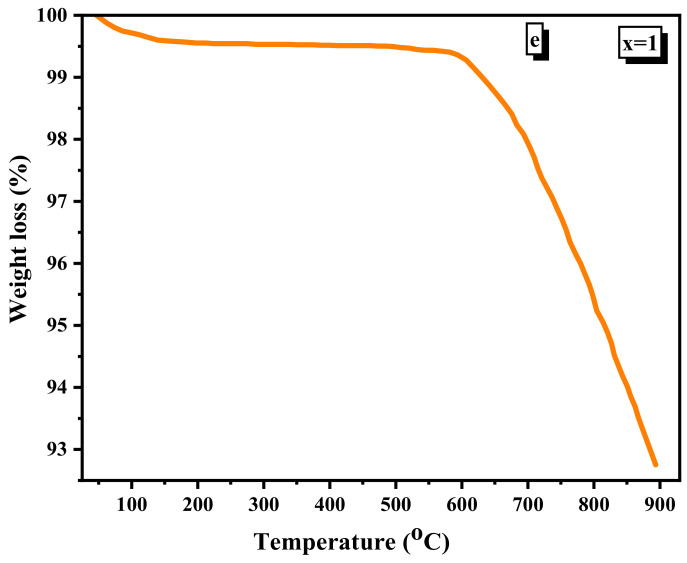
(**a**) TGA curve of ${\mathrm{Ni}}_{1-x}^{+2}{\mathrm{Mg}}_{x}^{+2}{\mathrm{Fe}}_{2}^{+3}O_{4}$ (x = 0). (**b**) TGA curve of ${\mathrm{Ni}}_{1-x}^{+2}{\mathrm{Mg}}_{x}^{+2}{\mathrm{Fe}}_{2}^{+3}O_{4}$ (x = 0.2). (**c**) TGA curve of ${\mathrm{Ni}}_{1-x}^{+2}{\mathrm{Mg}}_{x}^{+2}{\mathrm{Fe}}_{2}^{+3}O_{4}$ (x = 0.6). (**d**) TGA curve of ${\mathrm{Ni}}_{1-x}^{+2}{\mathrm{Mg}}_{x}^{+2}{\mathrm{Fe}}_{2}^{+3}O_{4}$ (x = 0.8). (**e**) TGA curve of ${\mathrm{Ni}}_{1-x}^{+2}{\mathrm{Mg}}_{x}^{+2}{\mathrm{Fe}}_{2}^{+3}O_{4}$ (x = 1).

Figure 8a–e shows the TGA curves of ${\mathrm{Ni}}_{1-x}^{+2}{\mathrm{Mg}}_{x}^{+2}{\mathrm{Fe}}_{2}^{+3}O_{4}$ (x = 0, 0.2, 0.6, 0.8, and 1) at temperatures changing from room temperature (RT) to 1173 K. The TGA curves indicated that weight loss happens in two stages. The first stage from room temperature to 373 K is due to H_2_O evaporation. In comparison, the second from 773 K up to 1073 K is due to the degradation and decomposition of the organic components that resulted during the preparation of the samples by the flash auto combustion method. Weight loss in the first stage is about 1%, while it is roughly 9% in the second stage. There is a shift from 723 K to 893 K when Mg concentration is increased in the samples, which implies that the temperature stability improves due to increasing the Mg concentration [44].

### 3.6. Magnetic Properties of the Prepared Samples

#### 3.6.1. VSM (Vibrating Sample Magnetometer) Discussion

**Figure 9:**
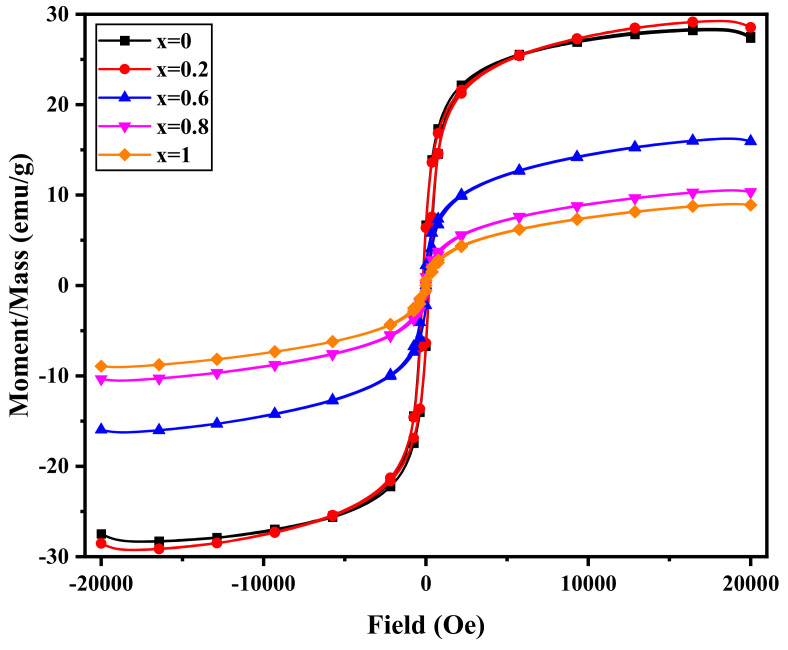
Magnetic hysteresis loops of ${\mathrm{Ni}}_{1-x}^{+2}{\mathrm{Mg}}_{x}^{+2}{\mathrm{Fe}}_{2}^{+3}O_{4}$ (x = 0, 0.2, 0.6, 0.8, and 1).

The magnetic hysteresis loop, which shows the magnetization vs. magnetic field for the ${\mathrm{Ni}}_{1-x}^{+2}{\mathrm{Mg}}_{x}^{+2}{\mathrm{Fe}}_{2}^{+3}O_{4}$ (x = 0, 0.2, 0.6, 0.8, and 1), is shown in Figure 9, which shows that the material has ferrimagnetic properties with a soft character. The magnetic parameters such as saturation magnetization (*M_s_*), remnant magnetization (*M_r_*), area of the hysteresis loop, coercive field (*H_c_*), squares, and magnetic moment can be deduced from the hysteresis loop.

The value of *M_s_*, *M_r_*, and *H_c_* decrease by increasing Mg concentration, as given in Table 6. The magnetic moment of Ni ions is 2*μ_B_* due to the two unpaired electrons in the outer shell.

In contrast, Mg ions have no unpaired electrons, and their magnetic moment equals zero. Additionally, Mg ions prefer to substitute for Ni ions at the B-site, which decreases the magnetization of the B-site, resulting in a drop in the net magnetization of the produced ferrite samples when the Mg content is increased. When some Mg ions occupy the A-site, it causes some shift of Fe^3+^ ions to the B-site with 5*μ_B_* magnetic moments. This migration of Fe^3+^ ions leads to an initial increase of *M_s_* at x = 0.2. So, we can say that two factors control magnetization in these nanoparticle samples. The first is the substitution of Mg instead of Ni at the B-site, the second is the occupation of Mg at the A-site, and the migration of Fe^3+^ from A to B-sites.

As given in Table 6, the *H_c_* decreases with increasing Mg content. The *H_c_* in nano-ferrites is influenced by many factors like crystallite size, canting angle, and anisotropy. Mg ions have zero orbital magnetic moments and zero spin angular moments compared with Ni and Fe^3+^, i.e., no anisotropy. By increasing Mg content, the magnetic anisotropy decreases, leading to the decrease of *H_c_* value. The prepared samples have a multidomain structure that permits *H_c_* reduction when the *D* reduces by increasing the Mg concentration. The non-saturation in the magnetic hysteresis loop can be attributed to several factors [17].

The *D* of the prepared samples is small, and it is equal to 12–19 nm, so the presence of superparamagnetic grains in these samples contributes to the non-saturation. On the other hand, the surface anisotropy of the small grain also contributes to the non-saturation of the magnetic moment. The *μ_exp_* (experimental magnetic moment) in Bohr magneton ($\mu_{B}$) was determined [44] using Equation (10):

(10) $$\mu_{exp}=\frac{M_{w}\times M_{s}}{5585\;}$$

The *μ_exp_* increases with increasing Mg content from x = 0 to x = 0.2 and decreases at higher Mg content, as given in Table 6. *μ_exp_* behaves identically to *M_s_*. The cation distribution may help to explain the prepared samples’ magnetization behavior. Neal’s theory was used to compute the net magnetic moment of the manufactured ferrite samples based on the X-ray cation distribution. Increased Mg concentration causes an increase in the X-ray magnetic moment, as seen in Table 6; this is owing to a rise in Fe^3+^ migration from B to A-site, which decreases net magnetization.

#### 3.6.2. Initial Permeability Discussion

**Figure 10:**
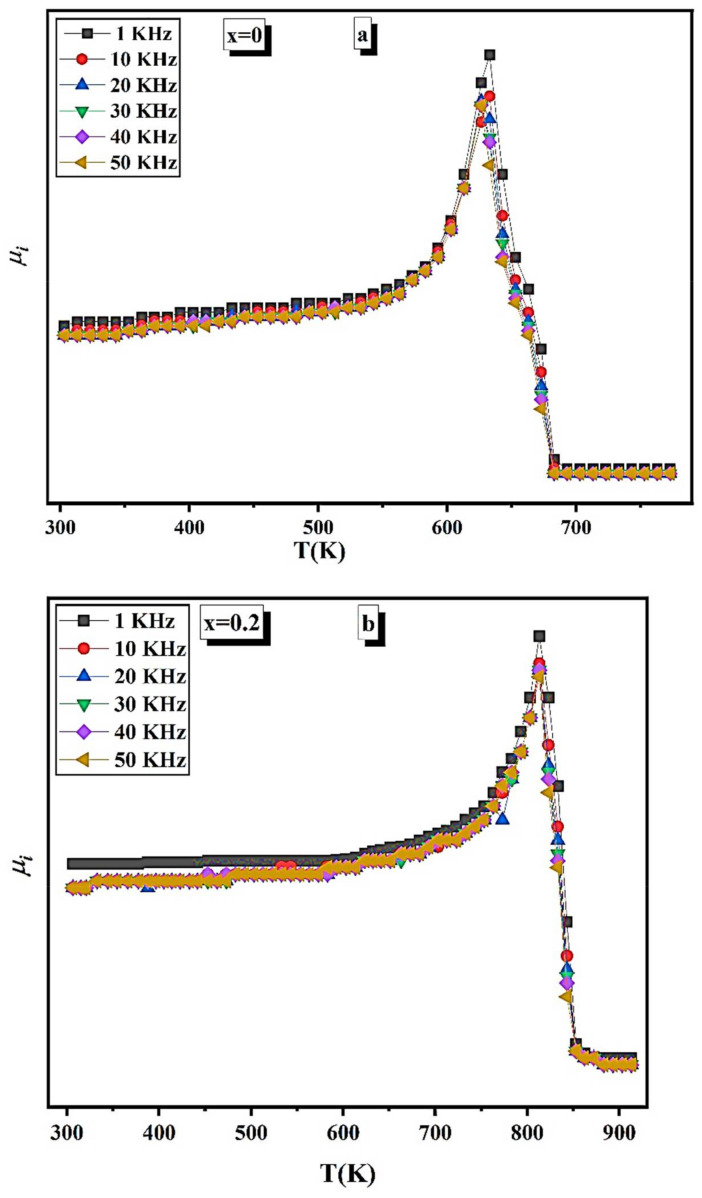

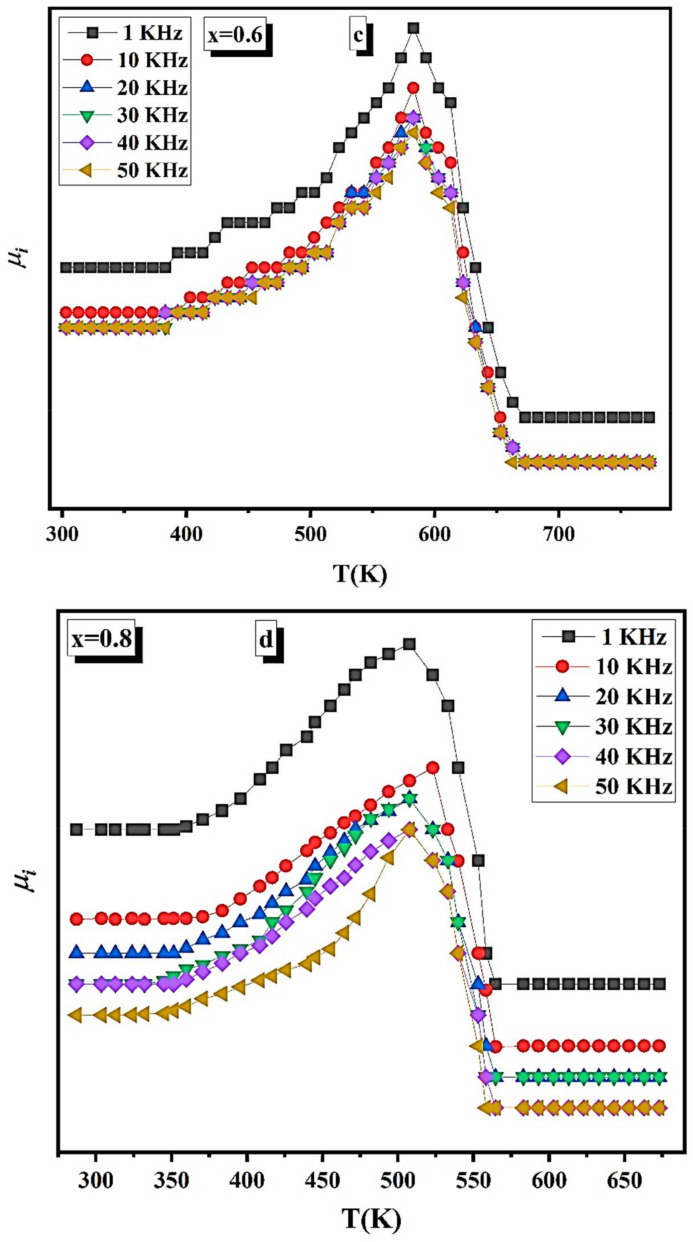

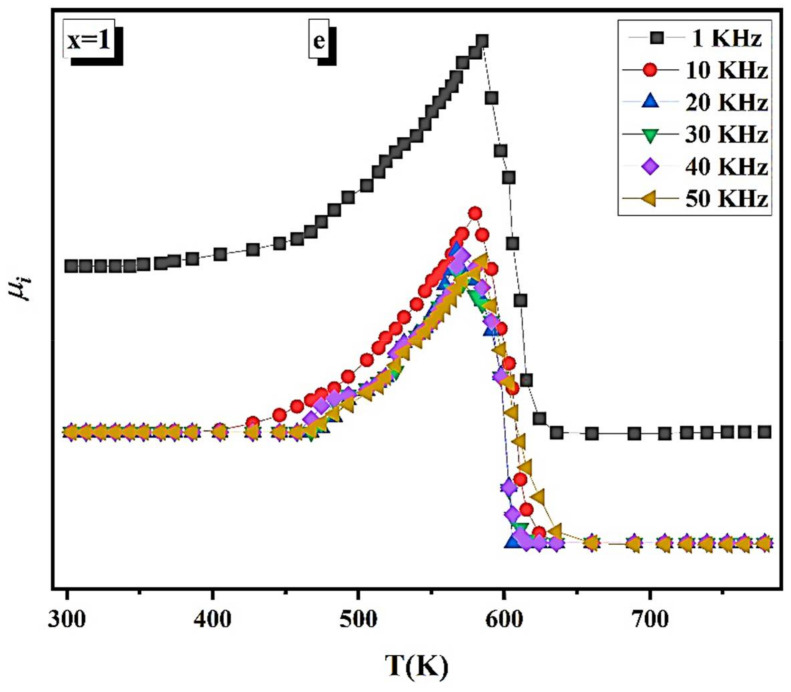
(**a**) *μ_i_* as a function of temperature at different frequencies of ${\mathrm{Ni}}_{1-x}^{+2}{\mathrm{Mg}}_{x}^{+2}{\mathrm{Fe}}_{2}^{+3}O_{4}$ (x = 0). (**b**) *μ_i_* as a function of temperature at different frequencies of ${\mathrm{Ni}}_{1-x}^{+2}{\mathrm{Mg}}_{x}^{+2}{\mathrm{Fe}}_{2}^{+3}O_{4}$ (x = 0.2). (**c**) *μ_i_* as a function of temperature at different frequencies of ${\mathrm{Ni}}_{1-x}^{+2}{\mathrm{Mg}}_{x}^{+2}{\mathrm{Fe}}_{2}^{+3}O_{4}$ (x = 0.6). (**d**) *μ_i_* as a function of temperature at different frequencies of ${\mathrm{Ni}}_{1-x}^{+2}{\mathrm{Mg}}_{x}^{+2}{\mathrm{Fe}}_{2}^{+3}O_{4}$ (x = 0.8). (**e**) *μ_i_* as a function of temperature at different frequencies of ${\mathrm{Ni}}_{1-x}^{+2}{\mathrm{Mg}}_{x}^{+2}{\mathrm{Fe}}_{2}^{+3}O_{4}$ (x = 1).

Figure 10a–e shows the variation of initial permeability (*μ_i_*) of ${\mathrm{Ni}}_{1-x}^{+2}{\mathrm{Mg}}_{x}^{+2}{\mathrm{Fe}}_{2}^{+3}O_{4}$ (x = 0, 0.2, 0.6, 0.8, 1) at different frequencies from 1 kHz to 50 kHz. The *μ_i_* increases with temperature up to a certain peak, i.e., Hopkinson peak (HP), which indicates the presence of a monodomain for the prepared samples. This peak has occurred near the Curie temperature of the material, which was determined from Figure 10a–e and tabulated in Table 7.

It was noted from Figure 10a–e that the *μ_i_* decreased by increasing the applied frequency. The origin of magnetic permeability is the domain wall motion. However, at these sizes, the NPs are single magnetic domains. The factors that affect permeability are saturation magnetization (*M_s_*), crystallite size (*D*), and magnetic anisotropy constant (*K*). The initial permeability can be computed [36] according to the relation:

(11) $$\mu_{i}=\frac{M_{s}^{2}\;D}{k};$$

The first two parameters (*M_s_* and *D*) do not affect the permeability. In contrast, the predominant factor is magnetic anisotropy. The HP humps may reduce the magnetic anisotropy field, which is faster than the decrease in *M_s_*.

The *μ_i_* sharply decreases at *T_C_*, making the prepared samples an extreme candidate for magnetic switch devices. The height of the HP peak decreases as the Mg content increases. Near *T_C_*, the samples transform to a paramagnetic state, leading to the decrease of *μ_i_*. Furthermore, the *μ_i_* increases by increasing the porosity, facilitating the domain wall motion.

The value of *T_C_* and the permeability change rate, i.e., the slope of the linear part of *μ_i_* vs. *T_C_*, is given in Table 7. The *T_C_* has a maximum at the x = 0.2, which is the same behavior as *M_s_*. At this sample x = 0.2, the material has a maximum magnetic exchange interaction. As the *T_C_* varies between 513 K and 813 K, it is simple to obtain magnetic material with the necessary *T_C_* by adjusting the Mg content.

The rate of permeability change with a temperature near the Curie point was calculated and given in Table 7. The higher the rate, the better the material can be used as a magnetic temperature transducer.

### 3.7. Electrical Properties of the Prepared Samples

#### 3.7.1. DC Resistivity Discussion

**Figure 11:**
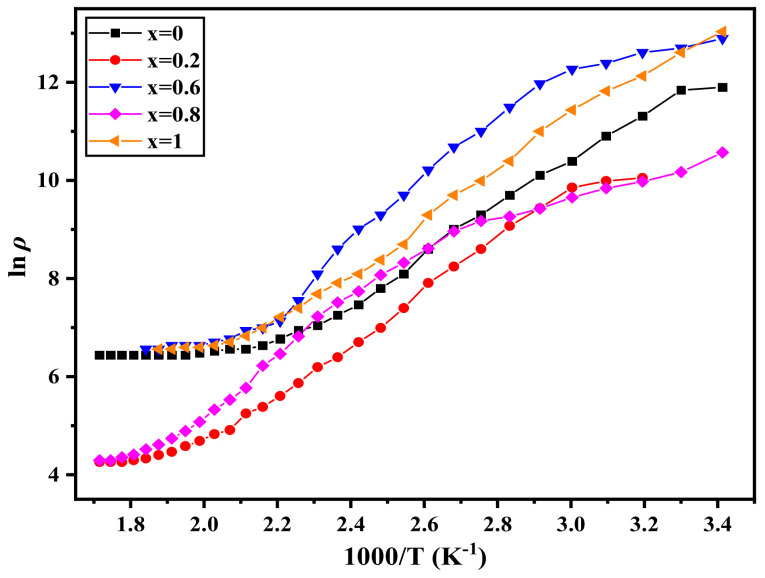
The variation of DC resistivity as a function of the reciprocal temperature of ${\mathrm{Ni}}_{1-x}^{+2}{\mathrm{Mg}}_{x}^{+2}{\mathrm{Fe}}_{2}^{+3}O_{4}$ (x = 0, 0.2, 0.6, 0.8, and 1).

The DC electrical resistivity (*ρ*) of the nano-ferrites system ${\mathrm{Ni}}_{1-x}^{+2}{\mathrm{Mg}}_{x}^{+2}{\mathrm{Fe}}_{2}^{+3}O_{4}$ (x = 0, 0.2, 0.6, 0.8, and 1) presented in Figure 11 was determined using the two-probe method as a function of reciprocal absolute temperature (1000/T). It is noted that a linear relationship exists between two regions and that it terminates at *T_C_*. This is the moment at which the ferrimagnetic state changes to paramagnetic. Increased magnesium content shifted the *T_C_* to a higher temperature. The increase in *T_C_* with increasing Mg concentration, as seen in Table 8, can be explained by the A–B exchange interaction, which is dependent on the Fe^+3^ distribution between A and B-sites. The first region is ferrimagnetic and conducted via hopping (hopping conduction mechanism). The second region is a paramagnetic area that belongs to the disordered state. The electrical resistance of all samples decreases with increasing temperature, confirming the semiconductor nature but not the semiconducting process. Numerous studies have also discovered that ferrite’s DC electrical resistivity is dependent on such a tendency [45,46,47]. The Verwey–de-Boer hopping process is used to describe the ferrite conduction mechanism [48], in which electrons hop between identical ions but in different valence states. Another aspect influencing resistivity behavior with temperature is thermally stimulated mobility of the charge carrier, which leads to a decrease in resistivity.

The activation energy (*E_a_*) was determined [49] using the following equation from the temperature dependence of resistivity (*ρ*):

(12) $$ln\rho =ln\rho_{0}+\frac{E_{a}}{K_{B}\;T};$$

where *ρ*_0_ is the pre-exponential factor, the *E_a_* increases from 0.27 eV to 0.53 eV as the Mg content increases, as illustrated in Table 8. *E_a_* behaves similarly to resistivity. The sample with high resistivity exhibits high activation energy. The *E_P_* is higher than the *E_f_* (the activation energy for the ferrimagnetic state), which indicates the influence of magnetic order on the conduction behavior. The values of *E_a_* show the presence of a hopping conduction mechanism.

#### 3.7.2. Dielectric Constant (The Real Part of Permittivity) Discussion

**Figure 12:**
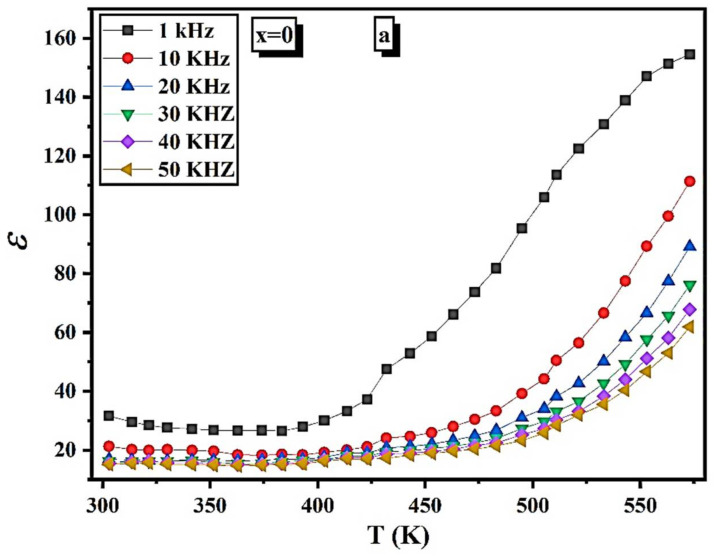

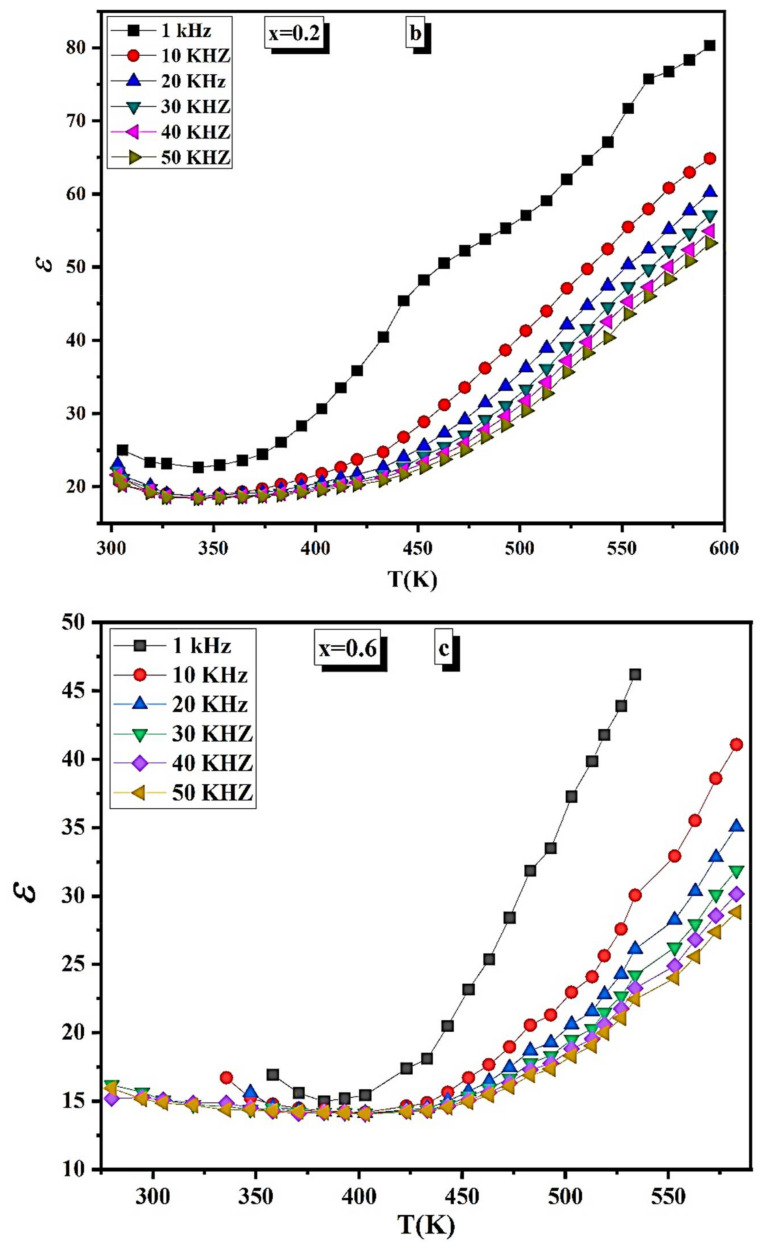

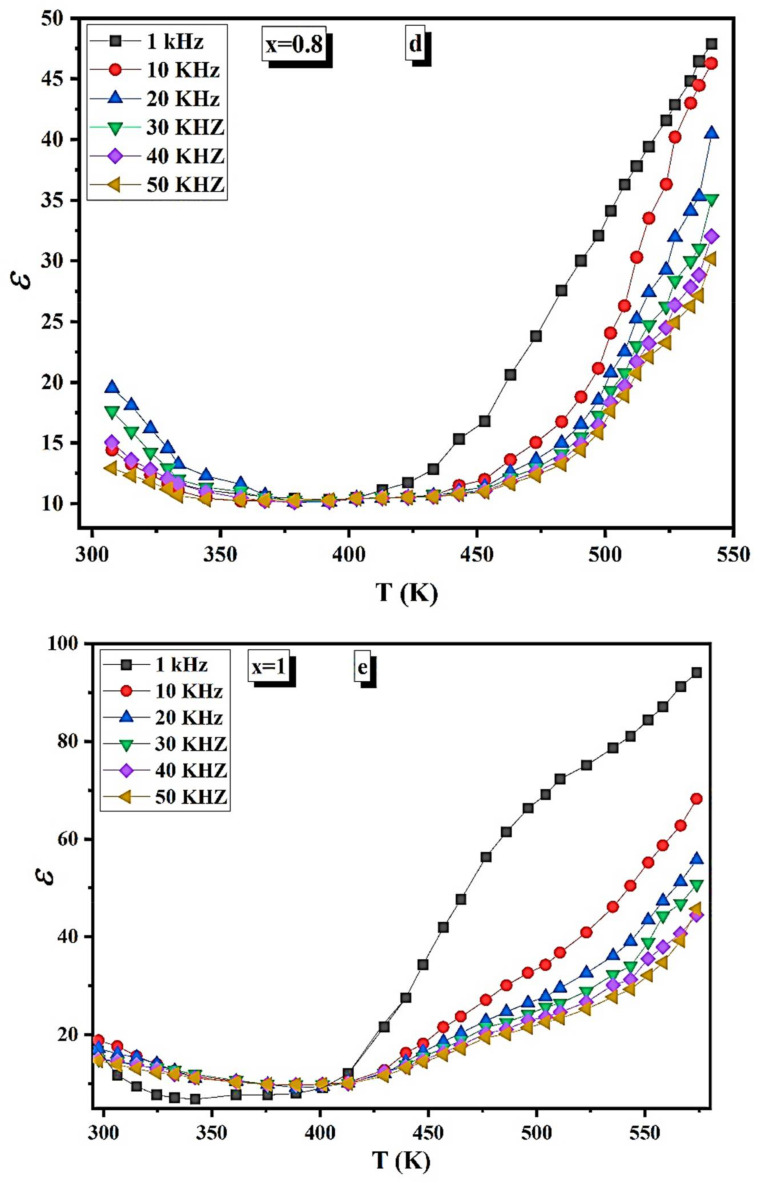
(**a**) *ε* as a function of the temperature of ${\mathrm{Ni}}_{1-x}^{+2}{\mathrm{Mg}}_{x}^{+2}{\mathrm{Fe}}_{2}^{+3}O_{4}$ (x = 0). (**b**) *ε* as a function of the temperature of ${\mathrm{Ni}}_{1-x}^{+2}{\mathrm{Mg}}_{x}^{+2}{\mathrm{Fe}}_{2}^{+3}O_{4}$ (x = 0.2). (**c**) *ε* as a function of the temperature of ${\mathrm{Ni}}_{1-x}^{+2}{\mathrm{Mg}}_{x}^{+2}{\mathrm{Fe}}_{2}^{+3}O_{4}$ (x = 0.6). (**d**) *ε* as a function of the temperature of ${\mathrm{Ni}}_{1-x}^{+2}{\mathrm{Mg}}_{x}^{+2}{\mathrm{Fe}}_{2}^{+3}O_{4}$ (x = 0.8). (**e**) *ε* as a function of the temperature of ${\mathrm{Ni}}_{1-x}^{+2}{\mathrm{Mg}}_{x}^{+2}{\mathrm{Fe}}_{2}^{+3}O_{4}$ (x = 1).

The temperature behavior of the dielectric constant (*ε*) was studied from room temperature up to 550 K at different frequencies (1–50 kHz, Δ = 10 kHz,) for all the prepared samples. The changing of *ε* with temperature is represented in Figure 12a–e.

The *ε* increases very slowly up to 400 K, and above this temperature, the *ε* rises rapidly. Furthermore, it is found that the *ε* decreases with increasing frequency. According to Koop’s model, *ε* is inversely proportional to the resistivity’s square root. As a result, the increase in *ε* is projected as the temperature rises. The value of *ε* increases dramatically when the temperature approaches the *T_C_* of ferrite, as seen in Table 8. For the ferrites, the source of the *ε* is due to four polarization types [50,51], which are interfacial (P_i_), dipolar (P_d_), atomic (P_a_), and electronic (P_e_). At low temperatures, the four types of polarization contributed to the *ε*, whereas the rapid increase of *ε* with increasing temperature is primarily due to the P_i_ and P_d_, which are temperature-dependent [50]—the P_i_ results from charge accumulation at grain boundaries. The P_e_ is increased by increasing the temperature at which the hopping electrons are thermally activated, which requires a quick increase in the dielectric constant. The intriguing dielectric properties of oxide composites have recently been reported and analyzed [52,53,54].

Table 9 shows the effect of Mg content on *ε* at a fixed temperature of 500 K and a frequency of 10 kHz. The maximum *ε* value is at x = 0.2, making this sample a candidate for capacitor fabrication. After x = 0.2, the *ε* decreases up to the sample x = 0.6 and increases with increasing Mg concentration. We can say that the conduction mechanism is similar to the polarization mechanism in ferrite. The local displacement arises from the hopping electron between adjacent octahedral ions in the spinel lattice, contributing to electric polarization and dielectric constant.

## 4. Conclusions

The flash auto combustion approach was used to create nano-ferrites ${\mathrm{Ni}}_{1-x}^{+2}{\mathrm{Mg}}_{x}^{+2}{\mathrm{Fe}}_{2}^{+3}O_{4}$ (x = 0, 0.2, 0.6, 0.8, and 1). XRD and TEM proved the single-phase cubic spinel. Images of the SEM micrographs of each sample reveal a consistent grain growth in terms of particle size. The EDX images shown alongside SEM contain no impurity peaks. The *F_tetra_* drops when the Mg content increases, whereas the *F_octa_* decreases when the Mg content increases to x = 0.2 and then increases. TGA curves have two weight loss regions. The first stage ranges from RT up to 373 K, and the second stage ranges from 773 K up to 1073 K. The weight loss for the first region is equal to 1%, whereas, for the second region, it is equal to 9%. It can be said from the VSM results that the studied samples have multidomain structure (superparamagnetic grains which contribute to the non-saturation), which permits the decrease of *H_c_* when the crystallite size decreases by increasing Mg content. The *T_C_* changes from 513 K to 813 K, so the desired magnetic materials can be easily obtained with the required Curie temperature simply by changing the Mg content. The electrical resistance of all samples decreases with increasing temperature, confirming the semiconductor nature. The obtained values of *E_a_* suggest the existence of a hopping conduction mechanism. The ${\mathrm{Ni}}_{1-x}^{+2}{\mathrm{Mg}}_{x}^{+2}{\mathrm{Fe}}_{2}^{+3}O_{4}$ samples that recorded the higher rate of permeability change with a temperature near the Curie point are candidates for use as magnetic temperature transducer devices.

## Figures and Tables

**Table 1:** X-ray cation distribution, ionic radius of the (A) position ($r_{A}$), ionic radius of the (B) position ($r_{B}$), oxygen positional parameter (u), inversion parameter (δ), and bond length of A-site ($R_{A}$) and B-site ($R_{B}$) for ${\mathrm{Ni}}_{1-x}^{+2}{\mathrm{Mg}}_{x}^{+2}{\mathrm{Fe}}_{2}^{+3}O_{4}$ (x = 0, 0.2, 0.6, 0.8, and 1).

x	Cation Distribution		$r_{A}$ (Å)	$r_{B}$ (Å)	*u* (Å)	*δ* (Å)	Bond Length (Å)		$\mu_{th}$ $\;(\mu_{B})$
	A-Site	B-Site					$R_{A}$	$R_{B}$	
0	$\left({\mathrm{Fe}}_{1}^{+3}\right)$	(${\mathrm{Ni}}_{1}^{+2}{\mathrm{Fe}}_{1}^{+3})$	0.64	0.6650	0.3861	0.0111	1.9599	1.988	2.00
0.2	$\left({\mathrm{Mg}}_{0.004}^{+2}{\mathrm{Fe}}_{0.996}^{+3}\right)$	$\left({\mathrm{Mg}}_{0.196}^{+2}{\mathrm{Ni}}_{0.8}^{+2}{\mathrm{Fe}}_{1.004}^{+3}\right)$	0.6403	0.6670	0.3860	0.0110	1.9596	1.992	1.64
0.6	$\left({\mathrm{Mg}}_{0.012}^{+2}{\mathrm{Fe}}_{0.988}^{+3}\right)$	$\left({\mathrm{Mg}}_{0.588}^{+2}{\mathrm{Ni}}_{0.4}^{+2}{\mathrm{Fe}}_{1.012}^{+3}\right)$	0.6409	0.6730	0.3858	0.0108	1.9609	1.998	0.92
0.8	$\left({\mathrm{Mg}}_{0.016}^{+2}{\mathrm{Fe}}_{0.984}^{+3}\right)$	$\left({\mathrm{Mg}}_{0.784}^{+2}{\mathrm{Ni}}_{0.2}^{+2}{\mathrm{Fe}}_{1.016}^{+3}\right)$	0.6413	0.6760	0.3857	0.0107	1.9612	2.000	0.56
1	$\left({\mathrm{Mg}}_{0.02}^{+2}{\mathrm{Fe}}_{0.98}^{+3}\right)$	$\left({\mathrm{Mg}}_{0.98}^{+2}{\mathrm{Fe}}_{1.02}^{+3}\right)$	0.6416	0.6790	0.3856	0.0106	1.9615	2.011	0.2

**Table 2:** Crystallite size (D), theoretical X-ray density ($\rho_{x}$), bulk density ($\rho_{bulk}$), porosity (P), and crystallite size, lattice parameter, and theoretical magnetic moment from HRTEM for ${\mathrm{Ni}}_{1-x}^{+2}{\mathrm{Mg}}_{x}^{+2}{\mathrm{Fe}}_{2}^{+3}O_{4}$ (x = 0, 0.2, 0.6, 0.8, and 1).

x	*D* (nm)	$\rho_{x}$ (g/cm^3^)	$\rho_{bulk}$ (g/cm^3^)	*P* (%)	*D* (nm)	$a_{th}$ (Å)	$\mu_{th}\;(\mu_{B})$
	XRD				HRTEM		
0	21	5.359	3.759	29.85	20.92	8.310	2.00
0.2	19	5.126	3.621	29.36	17.64	8.319	1.64
0.6	14	4.825	3.357	30.42	13.79	8.335	0.92
0.8	12	4.723	3.158	33.13	14.62	8.343	0.56
1	17	4.586	3.161	31.07	17.70	8.351	0.20

**Table 3:** Average pores size (μm) for ${\mathrm{Ni}}_{1-x}^{+2}{\mathrm{Mg}}_{x}^{+2}{\mathrm{Fe}}_{2}^{+3}O_{4}$ (x = 0, 0.2, 0.6, 0.8, and 1).

x	Average of Pores Size (μm)
0	0.23
0.2	0.07
0.6	0.15
0.8	0.144
1	0.09

**Table 4:** *ν_1_* (the absorption spectra of the A-site) and *ν*_2_ (the absorption spectra of the B-site), *R’_A_* (the bond length) and *F* for ${\mathrm{Ni}}_{1-x}^{+2}{\mathrm{Mg}}_{x}^{+2}{\mathrm{Fe}}_{2}^{+3}O_{4}$ (x = 0, 0.2, 0.6, 0.8, and 1).

x	*ν_1_*	*ν*_2_	$\;R^{’}{}_{A}$	*F_tetra_* (dyne/cm) × 10^5^	*F_octa_* (dyne/cm) × 10^5^
0	589	405	1.9599	2.54	1.20
0.2	580	382	1.9596	2.46	1.07
0.6	576	396	1.9609	2.43	1.15
0.8	574	403	1.9612	2.41	1.19
1	571	434	1.9615	2.39	1.38

**Table 5:** Deconvolution parameter of the FTIR spectra of the prepared ferrites, where (C) is the component band center, and (A) denotes the relative area of the component band.

		x = 0	x = 0.2	x = 0.6	x = 0.8	x = 1
I	**C**	393	358	376	403	389
	**A**	19.00	22.00	17.00	12.00	19.00
II	**C**	574	579	588	576	585
	**A**	16.00	29.00	34.00	42.00	28.00
III	**C**	1068	1099	1090	1112	974
	**A**	0.77	1.30	1.60	2.30	1.50
IV	**C**	1271	1279	1269	-	-
	**A**	1.30	25.00	5.00	-	-
V	**C**	1401	1412	1399	1411	1425
	**A**	4.40	3.70	2.30	4.10	19.00
VI	**C**	1554	1543	1599	1552	1554
	**A**	4.20	13.00	25.00	2.10	22.00
VII	**C**	1634	1627	-	1629	1638
	**A**	1.20	2.50	-	3.00	4.00
VIII	**C**	2105	2021	2111	2064	2123
	**A**	24.60	10.00	8.30	3.00	2.70
IX	**C**	2996	2346	2559	2911	-
	**A**	18.90	9.00	5.10	2.60	-
X	**C**	3433	3380	2981	3372	3403
	**A**	9.60	8.00	1.90	29.00	12.00

**Table 6:** The magnitudes of saturation magnetization (*M_s_*), coercivity (*H_c_*), remnant magnetization (*M_r_*), and experimental and theoretical magnetic moments (*µ_exp_*, *µ_th_*) of ${\mathrm{Ni}}_{1-x}^{+2}{\mathrm{Mg}}_{x}^{+2}{\mathrm{Fe}}_{2}^{+3}O_{4}$ (x = 0, 0.2, 0.6, 0.8, and 1).

x	*M_s_* (emu/g)	*H_c_* (Oe)	*M_r_* (emu/g)	*µ_exp_* _(_ *µ_B_*_)_	*µ_th_* _(_ *µ_B_*_)_
0	28.385	192.60	6.72	1.1912	2.00
0.2	29.256	178.96	6.39	1.1917	1.64
0.6	16.252	111.73	2.22	0.6219	0.92
0.8	10.527	95.462	0.96	0.3899	0.56
1	9.0194	88.344	0.58	0.3229	0.2

**Table 7:** The Curie temperature (*T_C_*) determined from the initial permeability data, permeability change rate d*µ_i_*/dT, and grain size of ${\mathrm{Ni}}_{1-x}^{+2}{\mathrm{Mg}}_{x}^{+2}{\mathrm{Fe}}_{2}^{+3}O_{4}$ (x = 0, 0.2, 0.6, 0.8, and 1).

x	*T_C_* (K)	Permeability Change Rate d*µ_i_*/dT, (K^−1^)	Grain Size (*D*), (nm)
0	633	2.243	84
0.2	813	1.740	71
0.6	584	0.977	97
0.8	513	0.514	125
1	614	0.447	136

**Table 8:** The Curie temperature (*T_C_*) determined from the DC electrical resistivity data and the activation energy for ferrimagnetic (*E_f_*) and paramagnetic (*E_P_*) of ${\mathrm{Ni}}_{1-x}^{+2}{\mathrm{Mg}}_{x}^{+2}{\mathrm{Fe}}_{2}^{+3}O_{4}$ (x = 0, 0.2, 0.6, 0.8, and 1).

x	*T_C_* (K)	*E_p_* (eV)	*E_f_* (eV)
0	656	0.27	0.39
0.2	666	0.40	0.43
0.6	686	0.53	0.40
0.8	705	0.51	0.19
1	666	0.41	0.34

**Table 9:** *ε* value at temperature 500 K and frequency of 10 kHz of ${\mathrm{Ni}}_{1-x}^{+2}{\mathrm{Mg}}_{x}^{+2}{\mathrm{Fe}}_{2}^{+3}O_{4}$ (x = 0, 0.2, 0.6, 0.8, and 1).

x	0	0.2	0.6	0.8	1
*ε*	40.42	40.93	22.71	24.16	33.74

## Data Availability

Not applicable.
